# The Emerging Role of Extracellular Vesicle-Mediated Drug Resistance in Cancers: Implications in Advanced Prostate Cancer

**DOI:** 10.1155/2015/454837

**Published:** 2015-10-26

**Authors:** Carolina Soekmadji, Colleen C. Nelson

**Affiliations:** Australian Prostate Cancer Research Centre-Queensland, Institute of Health and Biomedical Innovation, School of Biomedical Sciences, Faculty of Health, Queensland University of Technology, Translational Research Institute, Level 3 West, 37 Kent Street, Brisbane, QLD 4102, Australia

## Abstract

Emerging evidence has shown that the extracellular vesicles (EVs) regulate various biological processes and can control cell proliferation and survival, as well as being involved in normal cell development and diseases such as cancers. In cancer treatment, development of acquired drug resistance phenotype is a serious issue. Recently it has been shown that the presence of multidrug resistance proteins such as Pgp-1 and enrichment of the lipid ceramide in EVs could have a role in mediating drug resistance. EVs could also mediate multidrug resistance through uptake of drugs in vesicles and thus limit the bioavailability of drugs to treat cancer cells. In this review, we discussed the emerging evidence of the role EVs play in mediating drug resistance in cancers and in particular the role of EVs mediating drug resistance in advanced prostate cancer. The role of EV-associated multidrug resistance proteins, miRNA, mRNA, and lipid as well as the potential interaction(s) among these factors was probed. Lastly, we provide an overview of the current available treatments for advanced prostate cancer, considering where EVs may mediate the development of resistance against these drugs.

## 1. Introduction

Extracellular vesicles (EVs) are vesicles secreted by cells [1, 2], they are involved in mediating communication between cells by transferring signaling molecules, initiating a variety of cellular processes [2, 3]. The role of EVs in normal cellular growth and development has been reported as well as in the context of disease progression and cancer metastasis [4–6].

**Figure 1:**
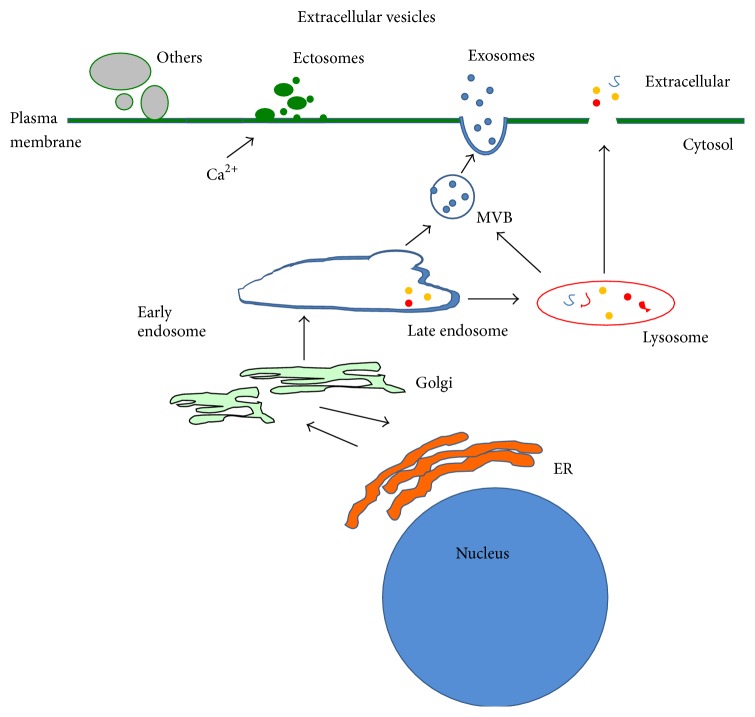
Schematic representative of ectosome and exosome secretion. Ectosomes or microvesicles are defined as extracellular vesicles which form through budding from plasma membrane. The exosomes are formed intracellularly, as proteins destined for exosome secretion are sorted in endosomes and packaged for exosome secretion through MVB pathways. Cross talk between MVB and endosomes has been reported as lysosomal markers such as LAMPs are found to be coisolated in secreted exosomes. Cells also secrete other types of vesicles such as oncosomes, prostasomes, and viral-like particles.

Exosomes and ectosomes are subclasses of secreted EVs; they have both been extensively characterized and shown to be functionally active in a number of studies [7–9]. The exosomes are nanosized vesicles, formed through intracellular budding at the multivesicular bodies (MVB). The biogenesis of exosomes is mediated by the action of Endosomal Sorting Complex Required for Transport (ESCRT) or by the lipid ceramide ([1, 2, 10], Figure 1). The MVB is an organelle involved in trafficking of vesicles from late endosomes to the plasma membrane, to mediate secretion [2, 11]. As such, exosomes contain MVB-associated proteins as well as RNAs encapsulated in a lipid bilayer with a specific composition of lipids, rich in sphingomyelin, cholesterol, and glycophospholipid [3, 12–14]. The ectosomes or microvesicles are vesicles bud from plasma membrane with a diameter up to 1 *μ*m. The ectosomes also have a specific lipid composition as they are enriched in phosphatidylserine. The exposure of phosphatidylserine on the cell surface is a characteristic of ectosome secretion [15]. Ectosome biogenesis is triggered by plasma membrane activation including intracellular calcium influx, mediated by ARF-6 and interactions between cytoskeletal resident proteins actin and myosin [1, 9, 16]. Others such as oncosomes, prostasomes, exosome-like vesicles, and viruslike vesicles are also members of EVs [2, 17]. The oncosomes are newly characterised EVs and are large vesicles (~0.8 *μ*m) which bud at the plasma membrane by “amoeboid” migratory cancer cells [18].

Understanding EV biogenesis has helped to differentiate exosomes and ectosomes; however classical ultracentrifugation and density gradient methodologies could not purely separate the subclasses of EVs, in particular to separate exosomes from other members of the EVs [2, 19]. Emerging evidence has shown the diversity of EVs; in particular, the previously characterized exosomes are not a homogenous population of vesicles [8]. As such, it was proposed to use the term EVs to include all secreted vesicles, even though classification based on biogenesis is still used in various publications [1, 2, 20].

Studies have explored EVs as potential biomarkers for various diseases, as the protein and RNA content of EVs has been shown to resemble the cell of origin [21, 22]. EV-derived protein glypican-1 has been shown to specifically indicate the presence of pancreatic cancer, indicating the real potential of EVs as a cancer biomarker [23]. Recently, it is becoming more accepted that EV secretion is not always constitutive as originally thought but is likely to be a cellular response toward changes in their environment. Exposure of cells to an effector can alter the EV content and their secretion process. For example, changes in cellular pH levels and exposure of cells to hypoxia growth conditions can alter the EV secretion [24, 25].

EVs have also been looked at as natural delivery agents for several treatments. By exploiting the “natural” path for vesicle endocytosis, EVs can bypass the immune system with no or low cytotoxic effects [26, 27]. This has been tested in melanoma cancer, as treatment of cancer with EVs containing the MAGE3 peptide has shown promising results, with no grade II toxicity [28]. EVs have been reported to internalize into cells through clathrin mediated endocytosis and micropinocytosis in PC12 cells [29] and in KRas expressing MiaPaCa-2 cells [30]. The cytoskeleton seems to play a role in this process [31]. However, due to the heterogenous nature of EVs, other mechanisms such as caveolin-dependent and caveolin-independent endocytosis and lipid raft mediated endocytosis were also observed [32–34]. As a shuttle mechanism between cells, EVs have been demonstrated to transfer prion and tau proteins as well as prion RNA [35, 36]. The transmission of prion protein (PrP) shows the role EVs play in the mechanism of transmissible neurodegenerative disorders [37].

In this review, we will discuss and assess the recent developments in the reported role of EVs in mediating drug resistance in cancer. We then discuss the potential role of how EVs could be involved in mediating the progression of prostate cancer to a more advanced, castrate resistant form of the disease. It is beyond the scope of this review to discuss in detail the many mechanisms of drug resistance; in-depth reviews on this topic have been published and are referred to in this paper.

## 2. The Emerging Role of EVs in Mediating Drug Resistance in Cancers

Acquired drug resistance is a major obstacle in cancer therapy. Drugs can be transported into target cells by diffusion, transport (by the action of transporters), or endocytosis (such as immunotoxins) [38]. Drug resistance accounts for cases of treatment failure by altering the absorption, metabolism, and/or efflux of drugs from the target cells. Drugs can also induce mutations of the targets and inhibit pathways for cellular apoptosis, leading to resistance. The development of new drugs with increased sensitivity and reducing patients' side effects toward treatment, while effective, can unfortunately induce the development of new pathways involved in drug resistance.

Cancer cells are capable of developing multidrug resistance (MDR). The acquired resistance toward a particular drug could lead to resistance toward other drugs with different structures and targets. Cancer cells do not respond equally towards drugs due to their distinctive genetic make-up and the expression of various oncogenes or tumor suppressor genes. Alteration of the cell cycle and checkpoints as well as expression of drug efflux pumps can contribute toward development of MDR [38, 39]. Changes in genetic or epigenetic factors and alteration in the interaction between host and tumor microenvironment also contribute toward drugs resistance [38, 39]. Tumor microenvironment can create a physical barrier, slowing the absorption of drugs by target cells and reducing the impact of oxygen radicals [40], thus reducing the effect of drugs in the body. In prostate cancer, chemotherapy drugs can prompt the secretion of WNT16B by the surrounding fibroblast cells, which then activates cellular survival Wnt pathways in prostate cancer cells, PC3 [41]. Furthermore, increasing evidence has shown that while drugs can cause apoptosis in the majority of a cell population, a small percentage of cells can survive [42, 43]. These cells, known as Cancer Stem Cells (CSC), express cell survival pathways BCL2/NF-*κ*B; they are able to self-renew and have pluripotent capacity [44–46]. The ability of CSC to self-renew is thought to drive its malignant phenotype and could be responsible for progression of cancer into a more advanced form of the disease.

Apart from their specific cell surface markers, CSC also express drug efflux pumps, the ABC drug transporters (ATP-binding cassette or multidrug resistance (MDR) protein) [47–49]. The characteristic expression level of ABC transporters in tumors has proposed the use of these proteins as biomarkers [50]. The ABC transporter protein superfamily is generally found at the plasma membrane and is recently shown to be present in EVs [51, 52]. Forty-eight members of ABC superfamily are found in humans [53, 54] including P-gp and multidrug resistance-associated protein (MRP) protein family. The ABC transporters maintain low intracellular drug concentrations and allow CSC to survive under therapy. It is hypothesized that MDR cell lines entrap drug molecules from their cytosol into acidic vesicles [55].

The multidrug resistance-1 (MDR1 or ABCB1) is the most characterized member of the ABC superfamily, also sometimes referred to as P-gp (P-glycoprotein). P-gp is able to transport a range of molecules, including amphipathic drug compounds, chemotherapy agents (such as doxorubicin), lipid, steroids, and peptides. P-gp activity is highly sensitive to its lipid environment [56]. The expressions of P-gp in cancers vary. For example, colon and kidney cancers have been shown to express high levels of P-gp, while ovary, breast, melanomas, lymphomas, lung, and multiple myelomas cancers express low levels of P-gp, even though P-gp expression elevates upon acquired drug resistance. Various strategies to overcome MDR by inhibiting the ABC transporters have been tested in phase III clinical trials with minimal success [57]; this may indicate that additional pathways are involved in the development of acquired MDR.

The members of ABC transporter family play a role in maintaining cellular homeostasis, by maintaining cellular lipid bilayers and transporting fatty acids and sterols in the body [58]. Chen et al. have reported that ABC transporters play a role in sequestration of drugs into intracellular organelles, as shown by accumulation of cisplatin in melanosomes [59], limiting the efficacy of drugs. It has been shown that MDR can also be mediated by changes in the membrane lipid ceramide [60]. The presence of P-gp has allowed the conversion of ceramide to sphingolipids, a major constituent of phospholipid membrane [61]. P-gp may be involved in the encapsulation of drugs into vesicles, which would then be exocytosed by cells [62]. P-gp has recently been shown to function through its association with F-actin and p-ERM [63].

Another study in pancreatic cancer has strengthened the benefit of targeting exosome pathways. In a pancreatic cancer cell model, GAIP interacting protein C terminus (GIPC, a regulator of G protein signaling) has been shown to modulate the exosome secretion and content. Interestingly, in the GIPC-depleted cells, the secreted exosomes contain high expressions of ABCG2, a member of the ABC transporter protein superfamily [64].

The presence of ABC transporters in exosomes has also been shown in breast and prostate cancers, where chemotherapy drug, docetaxel, can increase the presence of ABC transporters in secreted exosomes [51, 52, 65]. Docetaxel, a microtubule-targeting drug, decreases glucosylceramide synthase and sphingosine kinase-1 and increases ceramide synthase genes [66], confirming the potential to combine therapies by targeting ceramide and ABC transporters in a clinical setting. Cotargeting EV pathways would then be a promising avenue to overcome MDR in cancers, even though the pathways in which EV-derived ABC transporters can mediate MDR are relatively unexplored. Other molecules shown to mediate MDR, such as DeltaNp73 in colon cancer [67], have also been investigated for their role in mediating MDR through EVs such as exosomes.

The EV member ectosomes have also been shown as regulators for MDR. A study using ectosomes secreted by the adriamycin-resistant breast cancer cell line, MCF-7, can transfer Ca^2+^-permeable channels, TrpC5, to endothelial cells. The TrpC5 then stimulates the expression of P-gp through activation of the transcription factor NFATc3 (nuclear factor of activated T cells isoform c3), mediating the development of MDR in endothelial HMEC cells [68].

Apart from the presence of MDR-associated proteins, EVs can be involved in mediating resistance for drugs by sequestration of drugs in vesicles. It was reported in cisplatin-resistant melanoma cells that cisplatin is found in secreted exosomes [69]. Cisplatin is an alkylating agent that binds to DNA bases causing cross-links and breaks in DNA strands, interfering with DNA replication [70]. Encapsulation of cisplatin in exosomes is shown to be pH dependent. Accumulation of cisplatin has been shown in EVs when cells are grown in acidic extracellular pH rather than in neutral pH [69]. Exosome secretion and uptake are also enhanced in low pH [71], highlighting the importance of the extracellular environment in regulating the exosome secretion process.

## 3. The Emerging Role of EV Lipids in Mediating MDR

Recent reports have highlighted the role of EVs in regulating cellular lipid homeostasis. While exosome biogenesis is generally described as mediated by ESCRT protein complexes, Trajkovic et al. have shown that the lipid ceramide is able to regulate exosome secretion independently from the function of the ESCRT machinery [10]. Interestingly, ceramide, which is enriched in exosomes, is also known to mediate MDR [10]. The presence of ceramide in exosomes initially appears to be a downstream action of ceramide biosynthesis, as inhibition of neutral sphingomyelinases, enzymes which hydrolyse sphingomyelin to ceramide, can inhibit exosome secretion [10]. However, further studies in astrocytes have shown that ceramide in return can alter the exosome secretion, as treatment with extracellular ceramide stimulated exosome secretion in neutral sphingomyelinase 2 (nSMase2) deficient astrocytes cells. These cells do not naturally secrete exosomes; however, addition of extracellular C18 ceramide can stimulate exosome secretion in these cells [72].

Ceramide is a bioactive lipid; it has multiple signaling roles in endothelial cells, macrophages, and fibroblasts [73]. Ceramide accumulation can be mediated by various stimuli such as radiation [74], TNF-alpha [75], chemotherapy drugs [66], and natural anticancer agents (such as curcumin and capsaicin [76, 77]). Ceramide is capable of inducing apoptosis; cotreatment using P-gp antagonist and ceramides increases the rate of cellular death [61]. This process is mediated by alteration of mitochondrial membrane permeability through oligomerization of ceramide channels and reduction of prosurvival Bcl-2 proteins in mitochondria. The disruption of mitochondrial membrane permeability leads to generation of reactive oxygen species (ROS), which lead to apoptosis through caspase activation and mitogen-activated protein kinases (MAPKs)-dependent and -independent pathways [78, 79].

The exogenous ceramide could also induce secretion of ABC transporters in exosomes. For example, in breast cancer, C6 ceramide-induced secretion of breast cancer resistance protein (BCRP/ABCG2-associated exosomes) and inhibition of nSMase2 restored the cellular BCRP. This study suggests that lipid biosynthesis plays a role in recruiting ABC transporters into exosomes. Treatment with nuclear receptor antagonists, farnesoid X receptor antagonist guggulsterone and retinoid X receptor agonist bexarotene, increased the concentration of intracellular ceramide and reduced the BCRP protein in MDA-MB-231 cells [80], suggesting the role of ceramide-enriched exosomes in mediating MDR through ABC transporters.

Interestingly, a recent study has demonstrated that ceramide may not be the only lipid which is important in this process. A study using Synthetic Exosome-Like Nanoparticles, where the ratio of ordered lipids (lipid raft) versus disordered lipids (phospholipids) was equal to 6.0 (SELN6.0), inhibited the Notch-1 pathway in pancreatic cancer MiaPaCa-2 cells. The activation of NF-*κ*B leads to expression and the secretion of SDF-1alpha, a chemokine, which then activates the Akt survival pathway [81]. These accumulative findings, however, suggest that maintaining lipid homeostasis is also crucial in overcoming MDR in cancer therapy and that EVs could be important players in this process.

## 4. EV-Derived RNA: An Upstream Regulator of Acquired MDR?

While the exosomal lipids and ABC transporters in EV have emerged as important players in acquired MDR, exosomal RNA has recently been looked at as a player in this process. A recent study has shown that exosomal RNA could mediate MDR through interaction between stroma and cancers. Boelens et al. have reported the role of noncoding transcripts and transposable elements in stroma-derived exosomes in stimulating the activation of receptor RIG-I, which, in turn, activates the transcription factor STAT1-dependent signaling and Notch-3 in breast cancer cells, and accentuates the therapy resistance in tumor-initiating cells [82]. The EV-derived RNA may mediate MDR by regulating expression levels of drug efflux transporters or enzymes involved in lipid homeostasis, which would then disrupt the cellular signaling between the tumor and its microenvironment.

Reports have shown that exosomes-derived miRNAs are involved in MDR. The miR-222 increases the cellular survival in a docetaxel resistant breast cancer cell line MCF-7 [83]. A paper has also reported that secreted miR-221/222 in exosomes could mediate transfer of tamoxifen resistance in MCF-7 cells through downregulation of P27 and ERalpha [84]. In an ovarian cancer model, miR-21-3p has been shown to induce cisplatin resistance in A2780 cells, by targeting NAV3 gene [85].

While these accumulative findings strengthen the role of EV-derived miRNA, a report has also shown association between EV-derived mRNA levels and cancer. The mRNA levels of O(6)-methylguanine DNA methyltransferase and APNG (alkylpurine-DNA-N-glycosylase) are found to be enriched in tumor exosomes obtained from patients' blood and correlate with the level found in tumor cells [86], indicating the potential role on EV-derived mRNA in mediating MDR.

## 5. Treatment Options for Advanced Prostate Cancer

Prostate cancer is the most prevalent cancer in men worldwide and the second highest cause of cancer-related death in men after lung cancer [87]. Male hormones, androgens, are required for normal cell and cancer cell growth and maintenance. The androgens bind to the androgen receptor (AR) and are transported into the nucleus to initiate DNA transcription mechanisms.

**Figure 2:**
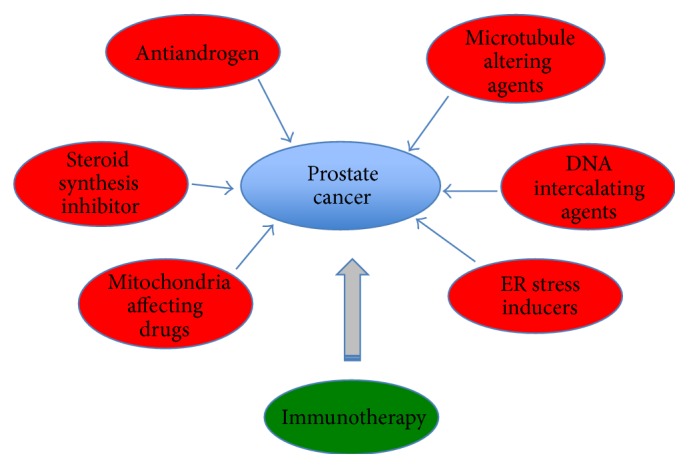
Current treatment options for advanced prostate cancer. Several classes of drugs (red circles) have been tested to target prostate cancer. These drugs inhibit various pathways known to regulate cancer proliferation and survival. A more personalized prostate cancer treatment has been developed through cancer immunotherapy (green circle), by activating patients' own cell mediated immunity against prostate cancer.

Removal of the prostate through surgery followed by radiation therapy and first-line androgen deprivation therapy for men with primary prostate cancer is suboptimal. Within two years, 25–40% of cases develop castrate resistant prostate cancer and continue to progress with metastatic disease [88]. Several classes of drugs/treatments have been developed to interfere with oncogenes/oncoproteins known to be involved in the progression of prostate cancer into a more advanced form of the disease (Figure 2; see also Table 1).

Targeting the AR has remained the main treatment for advanced prostate cancer. The AR is a steroid nuclear receptor; it is transcribed from the AR gene located on chromosome Xq11-12 [89, 90]. AR gene consists of eight exons, which encode four functional motifs: an amino-terminal domain, a DNA-binding domain (DBD), a hinge region, and a ligand-binding domain (LBD) [91–93]. The amino-terminal domain contains a transactivation domain, AF1, which is the primary transcriptional regulatory region. The LBD contains the secondary transcriptional regulatory region, AF2. The DBD is composed of two zinc fingers that are critical to DNA recognition and binding. The hinge domain of AR contains the nuclear localization signal which regulates the transactivation potential. The hinge domain is involved in intranuclear mobility of the AR and provides a site for binding of various androgen response elements as well as coactivators/corepressors [94].

Antiandrogens are commonly used in therapy to treat advanced prostate cancer. Androgen deprivation therapy (ADT) aims to limit the availability of androgens to bind to and activate AR, inhibiting the prostate cancer growth. ADT involves administering luteinizing-hormone-releasing hormone agonists or antagonists to disrupt the feedback loop within the hypothalamic gonadal axis, suppressing testosterone production by the testes. It has been reported that some prostate cancer cells are able to survive ADT and continue to maintain AR signaling [95–97]. AR antagonists, such as enzalutamide (MDV3100) or bicalutamide (Casodex), are designed to inhibit AR signaling by replacing the natural ligand DHT [98]. Bicalutamide (Casodex), enzalutamide predecessor, has shown some agonistic effect in cells which express high level of AR. Bicalutamide also increases AR recruitment to the enhancer region, thus increasing expression of AR regulated genes, such as PSA. Enzalutamide binds to AR with eightfold greater affinity than bicalutamide and only threefold less affinity than the natural ligand, DHT. MDV3100 also reduces the efficiency of AR translocation to the nucleus and impairs the binding of AR to the androgen response element and other factors that bind to AR [99]. In 2012, a new antiandrogen drug, ARN-509, with no observed agonistic effect was tested to treat castration-resistant prostate cancer (CRPC); it is currently being tested in phase II clinical trials [100, 101].

Docetaxel and its derivative, cabazitaxel, both belong to the taxane group and work by disrupting microtubule dynamics leading to mitotic arrest and apoptosis [103]. Cancer cells are usually rapidly dividing, requiring dynamic microtubule assembly during mitosis. Docetaxel stabilizes microtubules by binding to *β*-actin, thus inhibiting microtubule disassembly and mediating G2M arrest [122], and counteracts expression of oncogenes BCL-2 [104]. Prostate cancer is a relatively slow growing disease; thus, docetaxel acts by inhibiting AR nuclear translocation in androgen-dependent prostate cancer rather than stalling the cell cycle progression [105].

While treatment with docetaxel has been beneficial, 30% of CRPC patients who receive docetaxel therapy relapse [123]. Cancer cells can develop resistance to taxanes through mutations of the tubulin gene, expression of alternative tubulin isotypes, or drug efflux through multidrug resistant protein pumps [124]. Treatments combining docetaxel and Bcl-2 family inhibitors were tested in prostate cancer cell lines to overcome docetaxel resistance. ABT-263 and ABT-737 (Bcl-2, Bcl-xL, and Bcl-w inhibitors) enhanced the effect of docetaxel in inhibiting PC3 cell growth [125]. When given with prednisone, treatment with docetaxel every three weeks led to superior survival and improved rates of response in terms of pain, serum PSA level, and quality of life, as compared with mitoxantrone plus prednisone [126]. Cabazitaxel has recently replaced the mitochondria affecting drugs, such as mitoxantrone (discussed below), for prostate cancer patients who progress after docetaxel treatment. Cabazitaxel binds to different tubulin isotypes, human albumin, and lipoproteins and less efficiently to P-gp [127, 128].

Treatment combining ER stress inducers, bortezomib (Velcade), and docetaxel has been shown to benefit patients. Bortezomib is an inhibitor of the 20S proteasome, involved in ubiquitin-mediated protein degradation [110, 111]. Bortezomib modulates BCL-2 expression [104] but does not inhibit androgen mediated PSA mRNA expression. Bortezomib stimulates p53 translocation to the nucleus and enhances its DNA binding causing accumulation of p53-dependent transcripts in LNCaP pro5 cell line and leading to cell death [129].

Another ER stress inducer, estramustine (Emcyt), is a derivative of estrogen (estradiol) [130]. It has been shown to depolymerise cytoplasmic microtubules by binding to tubulin [112] and tau protein [113]. The microtubule and endoplasmic reticulum (ER) are interdependent, as prolonged disruption of microtubules will cause the ER to retract to the nucleus. This ER extension occurs at the same time as microtubule formation, by attaching to the growing end of microtubules and it is dependent on microtubule motor activity [131, 132].

Estramustine, used in combination with docetaxel, increases prostate cancer patients' median survival by two months in comparison with docetaxel alone [133]. In comparison with a combination of mitoxantrone and prednisone, the docetaxel and estramustine combination improves median survival by nearly two months for men with metastatic, androgen-independent prostate cancer [134]. To improve the synergistic effects, a combination of docetaxel, estramustine, and low-dose hydrocortisone has been trialed; unfortunately, 50% of men with CRPC acquired resistance toward treatments [135].

Satraplatin (JM-216) is an orally bioavailable platinum analog drug that has shown promise for treating advanced prostate cancer by mediating G2 M cell cycle arrest. Satraplatin is an analog of cisplatin and is effective in prostate cancer cells that are resistant to cisplatin [106, 107]. Satraplatin binds to DNA and creates a DNA adduct that is resistant to DNA nucleotide excision repair protein; it also evades DNA mismatch repair protein [108, 109].

Mitochondria affecting drugs, such as mitoxantrone (Novantrone), have also been tested in prostate cancer. Mitoxantrone blocks the cell cycling phase at S and G2 phase and is able to inhibit colony formation by prostate cancer cells* in vitro* [114]. Cells accumulate the drug within or near their mitochondria [115], increasing mitochondrial stress by depolarization of the mitochondrial membrane and causing release of cytochrome c into the cytosol, increased hydrogen peroxide production, activation of caspase cascades, and apoptosis [114]. Treatment with mitoxantrone also causes an increase in the expression of BCL-XI (a member of the BCL-2 family) and delays the onset of the p53 pathway. Both of these pathways are known to be involved in the survival of prostate cancer cells. BCL-2 is involved in mitochondrial membrane integrity and is involved in the Akt apoptosis pathway [136, 137]. Mitoxantrone also inhibits topoisomerase II-mediated DNA intercalation and generates free radicals that kill cancer cells [116].

Treatment by inhibiting* de novo* biosynthesis of androgens in CRPC has recently been investigated by targeting CYP17. CYP17 catalyzes two key steroid reactions involving 17alpha-hydroxylase and C(17,20)-lyase in the androgen biosynthesis pathway [117, 118]. The cytochrome P450 enzymes CYP11A1 and CYP17A1 are involved in intratumoral conversion of the weak adrenal androgens DHEA and androstenedione into the AR ligands testosterone and dihydrotestosterone [138]. CYP17A1 inhibitors, such as abiraterone acetate (Zytiga), are sought as a potential effective therapy for castrate resistance prostate cancer [117, 119]. However, a study in castration-resistant VCaP tumor xenografts shows development of abiraterone resistance. Abiraterone resistant prostate cancer cells express a T877A mutation in the AR, such as that found in LNCaP cells, which is activated by other steroids through CYP11A1-dependent pregnenolone/progesterone synthesis [138]. In a controlled COU-AA-302 study, abiraterone plus prednisone significantly prolonged median overall survival in chemotherapy-naive men with metastatic CRPC. Another CYP17 inhibitor, orteronel (TAK-700) with higher specificity for 17,20-lyase than for 17*α*-hydroxylase, has been developed to reduce abiraterone-induced toxicity. Orteronel is shown to reduce the expression of PSA (Prostate Specific Antigen), an AR regulated gene and current clinical biomarker for prostate cancer, in metastatic CRPC [120].

Both enzalutamide and abiraterone have been designed to further suppress AR signaling in CRPC. Resistance to enzalutamide and abiraterone is indicated by a return of activated AR signaling. This phenomenon may be mediated by AR splice variants (AR-Vs), in particular, variant 7 (AR-V7) [121]. The AR-V7 lacks the LBD, which is the target of enzalutamide and abiraterone. Interestingly, AR-V7 can remain constitutively active as a transcription factor [121]. Activation of mutant AR by eplerenone can be inhibited by antiandrogens (MDV3100, bicalutamide) or abiraterone. Treatment with abiraterone could overcome resistance caused by activation of AR by residual ligands or coadministered drugs [139]. Recently, a new drug, EPI-506, which targets the N-terminal of AR was announced to be tested in prostate cancer patients [102].

Personalized treatment by implementing cancer immunotherapy is being developed for prostate cancer. Sipuleucel-T (APC8015 or Provenge) is a novel cancer immunotherapy, which utilizes a recombinant fusion protein (PA2024) consisting of granulocyte macrophage colony-stimulating factor and prostatic acid phosphatase to stimulate the patient's T cells response [140]. Sipuleucel-T has shown a positive effect in extending survival by median 4.1 months in men with metastatic CRPC (IMPACT phase III trial data) [141], while combination with docetaxel does not seem to enhance the efficacy of this treatment [142].

Recently, a new phase 1/2 clinical trial has been approved to evaluate the combination of immunotherapy ADXS-PSA and pembrolizumab. The ADXS-PSA (ADXS31-142) is an investigational* Listeria monocytogenes*- (Lm-) LLO immunotherapy to reduce the immunosuppressive activity of myeloid-derived suppressor cells and regulatory T cells in the tumor microenvironment. Pembrolizumab (Keytruda) is a humanized monoclonal antibody that blocks the interaction between programmed death receptor-1 and its ligands. Combinations of both agents will be tested in metastatic CRPC patients [143].

## 6. Can EVs Mediate Drug Resistance in Prostate Cancer?

Drug resistance is a major problem in the treatment of many cancers, including prostate cancer. Various roles of EVs in prostate cancer have been reported; EVs can alter the changes of tumor fibroblasts to myofibroblasts, as well as promoting angiogenesis in the tumor microenvironment [144, 145]. Amoeboid prostate cancer can also secrete oncosomes upon treatment with growth factors, which are involved in cancer cell migration, invasion, and metastasis [17]. EVs have also been looked at as a potential source of biomarkers in prostate cancer [146, 147], while their role in mediating MDR in prostate cancer has just started to be investigated.

Normal prostate cells express BCRP (ABCG2) and MRP (ABCC1) [148, 149] but not P-gp (ABCB1) [149], whereas prostate cancer cells do express P-gp [150]. Corcoran et al. have shown that exosomes can mediate transfer of docetaxel resistance through P-gp transport via EVs in a DU145 cell line [51], even though a mechanism of how exosomal-derived P-gp could mediate drug resistance was not described.

In a non-EVs study, the contribution of P-gp in mediating drug resistance in prostate cancer was previously reported. In a study, prostate cancer cell lines were cotreated with doxorubicin (a gamma topoisomerase poison, which causes DNA damage) and drugs that are known to partially target the MDR in tumors (verapamil, cyclosporine A, and tamoxifen) [151]. Verapamil acts by inhibiting voltage-gated K+ channels, which then inhibit the proliferation of LNCaP cells [152]. The cotreatment resulted in a synergistic effect in PC-3 and DU-145 cells, but not LNCaP cells, indicating the role of P-gp in PC-3 and DU145 resistance. However, the author has also reported that low MDR-reversal rates could suggest that an alternative MDR-independent pathway may be responsible for triggering drug resistance in prostate cancer [151].

Lipid homeostasis seems to play a major role in drug resistance in other cancers as discussed above. Disruption of lipid homeostasis such as treatment with ceramide can also be utilized to target prostate cancer. Ceramide can enhance activation of the intrinsic apoptotic pathways and enhance cell death induced by TNF-alpha in LNCaP cells [153]. Treatment with bioactive ceramide also induces apoptosis in AR expressing LNCaP prostate cancer cells by causing nuclear fragmentation and activation of c-Jun N-terminal kinase (JNK), independent of PKC pathways [79]. In AR negative prostate cancer cells PC3, ceramide activates the protein phosphatase 2A (PP2A) and increased p27(kip1) protein levels via Akt-dependent and Akt-independent pathways [154], suggesting some overlaps in pathways influenced by lipid homeostasis. A natural agent, capsaicin, mediates cell death through sphingomyelin hydrolysis by nSMase, to generate ceramide via the ERK and JNK pathways, resulting in cellular apoptosis in prostate cancer [155]. Alteration of membrane properties could lower the level of resistance [156], an area where EVs could contribute.

In a study on docetaxel resistance in prostate cancer, miR-34a shows clinical relevance. miR-34A could alter cellular response to docetaxel in prostate cancer cells through BCL-2, which then target SNCA and SCL7A5 [157]. Treatment of DU145 cells with fludarabine increase the secretion of exosome-associated miRNA. While the exosome-associated miRNA was not identified, fludarabine treatment is shown to retain the miR-485-3p in parental cells, which could play a role in resistance through regulation of the transcriptional repressor nuclear factor-Y which regulates transcription of topoisomerase IIalpha, multidrug resistance gene 1, and cyclin B2 prosurvival genes [158].

## 7. Conclusion

Prostate cancer is an extremely heterogeneous disease; the underlying mechanisms for progression to CRPC are complex [138, 159]. Exposure to drugs will prompt tumor cells to secrete proteins to the external environment, where these proteins mediate interaction with stroma, macrophages, dendritic cells, and others. Understanding factors that contribute toward MDR will help patients and clinicians to decide on the most suitable treatment regime for an individual patient, increasing the efficacy of drugs, while reducing unnecessary side effects.

The release of EVs is exacerbated in tumors leading to their increased presence in bodily fluids of cancer patients [160–162]. Tumor-derived EVs express an array of antigens that reflect the originating tumor cells, small RNAs, and specific composition of lipid bilayers. EVs secreted by a tumor can trigger an immune response and cellular differentiation in the tumor microenvironment and they have also been shown to support tumor escape and growth [144, 145, 163]. The presence of P-gp and ceramide in EVs indicates the potential role of EV in mediating MDR, even though the mechanisms by which P-gp and ceramide-derived EVs can mediate MDR in prostate cancer are not clear. The role of EVs in mediating MDR could be limited to drugs that alter the P-gp expression or functions or the lipid ceramide. Understanding the effect on EV pathways of various drugs used to target prostate cancer will help us to understand the role of EVs in mediating MDR in prostate cancer.

## Figures and Tables

**Table 1:** Classes of drugs used to treat advanced prostate cancer in the clinic.

Classes	Drugs	Mechanism(s)	References
Antiandrogens	(i) Enzalutamide (ii) Bicalutamide (iii) EPI-506 (iv) ARN-509	Inhibition on the activity of androgen receptor or its splice variant(s) in mediating DNA transcription	[95–102]

Microtubule altering agents	(i) Docetaxel (ii) Cabazitaxel	Disruption of microtubule; inhibiting AR translocation to nucleus; counteracting expression of oncogenes BCL-2	[103–105]

DNA intercalating agents	(i) Cisplatin (ii) Satraplatin	Platinum analog drug; creating a DNA adduct to allow DNA translation; overriding mechanism of DNA repair	[106, 107] [108, 109]

ER stress inducers	(i) Bortezomib (ii) Estramustine	Inhibition of the 20S proteasome; modulating BCL-2 expression; depolymerizing cytoplasmic microtubules by binding to tubulin and tau protein	[104, 110–113]

Mitochondria affecting drugs	Mitoxantrone	Mitochondria affecting drugs; causing mitochondrial stress by depolarization of the mitochondrial membrane; inhibiting topoisomerase II-mediated DNA intercalation	[114–116]

Steroid synthesis inhibitor	(i) Abiraterone (ii) Orteronel	Inhibiting *de novo* biosynthesis of androgens by targeting CYP17 in the androgen biosynthesis pathway; suppressing AR signaling in castrate resistant prostate cancer	[117–121]
